# Comparison of airway dimensions in skeletal Class I malocclusion subjects with different vertical facial patterns

**DOI:** 10.1590/2177-6709.22.6.035-042.oar

**Published:** 2017

**Authors:** Ana Paula Flores-Blancas, Marcos J. Carruitero, Carlos Flores-Mir

**Affiliations:** 1 Private practice (Trujillo, Peru).; 2 Universidad Privada Antenor Orrego, Facultad de Medicina Humana, Escuela Estomatología (Trujillo, Peru).; 3 University of Alberta, Division of Orthodontics (Edmonton, Canada).

**Keywords:** Upper airway, Vertical facial pattern, McNamara analysis, Vert index

## Abstract

**Objective:**

The aim of this study was to compare upper airway widths among skeletal Class I malocclusion subjects with different vertical facial patterns.

**Methods:**

The sample included a total of 99 lateral cephalograms of post pubertal individuals (18.19 ± 1.76 years old). The vertical facial pattern was determined by the Vert index. The McNamara method was used to quantify upper airway widths. ANOVA test and Student’s *t* test for independent groups were used, when normal distribution was not supported Kruskal-Wallis test and U-Mann-Whitney test were used. A multiple linear regression analysis was also performed.

**Results:**

Statistically significant differences in several nasopharyngeal widths were found among the distinct vertical facial patterns. Subjects with brachyfacial pattern presented larger nasopharyngeal widths than subjects with mesofacial (*p*= 0.030) or dolichofacial (*p*= 0.034) patterns. The larger the Vert value, the larger the nasopharyngeal widths (R^2^= 26.2%, *p*< 0.001). At the level of oropharynx no statistically significant differences were found.

**Conclusion:**

It was concluded that nasopharyngeal linear anteroposterior widths in Class I malocclusion brachyfacial are larger than in mesofacial and dolichofacial individuals. The Vert index only explained 25% of the total variability. No correlation was found for the oropharyngeal widths.

## RESUMO

**Objetivo:**

o objetivo desse estudo foi comparar as dimensões das vias aéreas superiores em indivíduos portadores de má oclusão de Classe I esquelética com diferentes padrões faciais verticais.

**Métodos:**

a amostra consistiu de 99 cefalogramas laterais de indivíduos na pós-puberdade (18,19 ± 1,76 anos). O padrão facial vertical foi determinado por meio do índice VERT. O método de McNamara foi utilizado para quantificar as dimensões das vias aéreas superiores. O teste ANOVA e o teste *t* de Student para grupos independentes foram utilizados e, quando a distribuição normal não era possível, o teste de Kruskal-Wallis e o teste U de Mann-Whitney foram aplicados. Foi também realizada uma análise de regressão linear múltipla.

**Resultados:**

diferenças estatisticamente significativas nas dimensões da nasofaringe foram encontradas entre os diferentes padrões faciais verticais. Os indivíduos com padrão braquifacial apresentaram dimensões nasofaríngeas maiores do que os indivíduos com padrão mesofacial (*p*= 0,030) ou dolicofacial (*p*= 0,034). Quanto maior o valor do VERT, maior a dimensão nasofaríngea (R^2^ = 26,2%, *p*< 0,001). Não foram encontradas, entretanto, diferenças estatisticamente significativas ao nível da orofaringe.

**Conclusão:**

pode-se concluir que as dimensões anteroposteriores lineares da nasofaringe nos indivíduos braquifaciais com má oclusão de Classe I são maiores do que nos indivíduos mesofaciais e dolicofaciais. O índice VERT foi capaz de explicar apenas 25% da variabilidade total. Não foi encontrada correlação para as dimensões da orofaringe.

## INTRODUCTION

Orthodontics has been long interested in the association between mode of breathing and craniofacial growth.^1^ ^-^ ^3^ The pharyngeal structures play an important role during breathing and swallowing functions. The pharynx can be anatomically separated in nasopharynx and oropharynx. It has been proposed that they may vary in dimensions based on orthopedic therapy^4^ or craniofacial growth.^5^ ^,^ ^6^

Morphological upper airway obstructive processes are factors that can lead into a partial or total upper airway obstruction. When that happens the resulting functional imbalance could lead into a significant mouth-breathing pattern, which may alter the craniofacial morphology and dental arch shape, ultimately producing a malocclusion.^7^ ^-^ ^11^

The relative growth and size of the soft tissues surrounding craniofacial skeletal structures determine the size of the pharyngeal space. The depth of the nasopharynx increases as its posterior wall becomes narrower.^9^ There is also a natural and anatomical predisposition of airway to become thinner as it has been suggested that subjects with Class I and Class II malocclusions and vertical growth patterns have significantly narrower upper pharyngeal airways than those with normal growth patterns.^12^ If the upper airway becomes narrower, in some cases the air flow resistance may increase, which may also increase the risk of snoring and, in severe cases, lead to obstructive sleep apnea.^11^ ^,^ ^13^ ^,^ ^14^

Altered upper airway function during the active period of craniofacial growth can also have a profound influence on the different facial growth patterns.^15^ ^,^ ^16^ In contrast, craniofacial anomalies, including maxillary or mandibular retrognathism, short mandibular body, clockwise rotation of the jaw, high palatal vault, narrow maxilla, and increased anterior face height have been associated with reduced pharyngeal airway.^15^ ^,^ ^17^ The usual unanswered question is which one may have occurred first.

For the assessment of the dimension of the airway, some linear distances have been described.^18^ ^,^ ^19^ Although the association between craniofacial morphology, breathing function and pharyngeal structures has been previously explored,^20^ ^-^ ^22^ its association with the vertical facial patterns has been assessed by using various methods to determine the facial pattern like, Y-axis,^13^ SN-Me angle^15^ or SN-GoGn angle;^23^ ^,^ ^24^ but not using the Vert index method, which relates both quantitative and qualitative facial vertical growth,^25^ being necessary a more insight in the field. 

The Vert index method is a method proposed by Ricketts which identify growth patterns taking into account five cephalometric measurements: facial axis, facial depth, mandibular plane, anteroinferior facial height, and mandibular arch. These values let classify the face into six types: severe brachyfacial, brachyfacial, mesofacial, light dolichofacial, dolichofacial, and severe dolichofacial^25^ (which can also be grouped into just three types: mesofacial, brachyfacial, and dolichofacial).^26^ ^,^ ^27^

The aim of this study was to compare the widths of the airways in Class I malocclusion subjects with different vertical facial patterns, by using the Vert index.

## MATERIAL AND METHODS

The present research was approved by the Stomatology Permanent Research Committee of the Antenor Orrego Private University (Trujillo, Peru).

### Study sample

This study was conducted on 99 lateral cephalograms of post pubertal individuals between 16 and 22 years old (18.19 ± 1.76): 52 women (16 - 22 years of age, 18.10 ± 1.91); and 47 men (16 - 21 years old age, 18.30 ± 1.59). The radiographs were randomly selected from all the available radiographs during the period 2010 to 2013 in a diagnostic imaging center in Trujillo, Peru. 

To determine the sample size, data from a pilot study with 24 radiographs was used. A statistical power of 80% and a confidence level of 95% was finally considered. The variance for brachyfacial individuals was 8.01 mm and for the mesofacial individuals was 6.98 mm. Therefore a minimum of 15 lateral cephalograms per facial vertical pattern was needed. Three groups of 31, 33, and 35 radiographs for each vertical facial pattern (mesofacial, dolichofacial and brachyfacial, respectively) were formed. 

The lateral cephalograms had the following selection criteria: cephalometric radiographs with adequate diagnostic contrast, with the same magnification, from patients with skeletal Class I relationship, no history of pharyngeal pathology based on clinical chart information, and with skeletal maturation at stage 6 of cervical vertebrae method.^28^

### Tracing

The cephalometric tracings, landmark identifications, and measurements were performed on acetate paper by one researcher.

**Figure 1:**
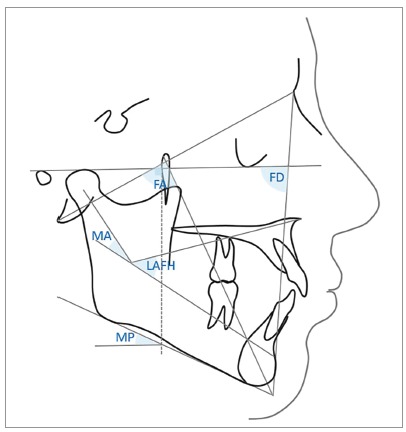
Angles for Vert index: FA, Facial Axis (angle between the lines Basion-Nasion and Gnation-Pterygoid); FD, Facial Depth (angle formed by lines Nasion-Pogonion and Porion-Orbitale); LAFH, Lower Anterior Facial Height, angle formed by lines ANS-Xi and Xi-MP; MP, Mandibular Plane (angle formed by lines Porion-Orbitale and Gonial-Menton); MA, Mandibular Arch (angle formed by lines Dc-Xi and Xi-MP).

The vertical facial pattern was determined by the Vert index.^25^ To determine the final vertical facial pattern, five cephalometric measurements were considered: [1] Facial Axis (FA), angle between the lines Basion-Nasion and Gnation-Pterygoid; [2] Facial Depth (FD), angle formed by lines Nasion-Pogonion and Porion-Orbitale; [3] Lower Anterior Facial Height (LAFH), angle formed by lines ANS-Xi and Xi-MP; [4] Mandibular Plane (MP), angle formed by lines Porion-Orbitale and Gonial-Menton, and [5] Mandibular Arch (MA), angle formed by lines Dc-Xi and Xi-MP (Fig 1). The facial type determined by the Vert index in adults is given by the following equation: {[(FA-90) / 3] + [(FD-90) / 3] + [(24,5-MP) / 4] + [(47-LAFH) / 4] + [(MA-28,5) / 4]} / 5. If the result was greater than +0.5, the patient was classified as brachyfacial; between -0.49 and +0.49, as mesofacial; and smaller than -0.5, as dolichofacial.

### Upper airway tracing

**Figure 2:**
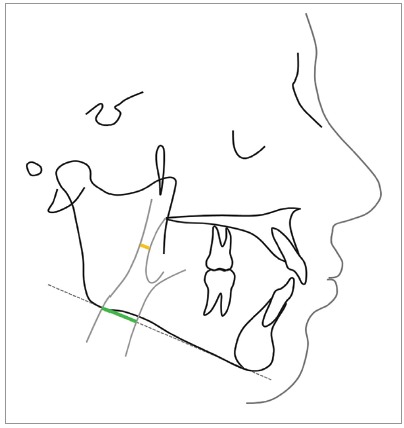
Airway widths according to McNamara analysis, nasopharynx (yellow line) and oropharynx (green line).

Nasopharynx (upper pharynx) and oropharynx (lower pharynx) tracings were made as proposed by McNamara.^18^ The width of the nasopharynx was measured linearly as the shortest distance from a point of the posterior wall of the palate to the posterior pharyngeal wall, parallel to the mandibular plane. The width of the oropharynx was evaluated at the point where, radiographically, the mandibular plane crosses the anterior and posterior pharyngeal walls (Fig 2).

### Method error

To evaluate the method error, measurements in 24 radiographic images not considered in the final study were considered. These measurements were carried out by the same researcher twice (the second time, after a week) in order to assess intra-examiner reliability for the cephalometric tracings. To assess the inter-examiners reliability, the same cases were evaluated by another researcher. The agreement between the observations of the nasopharynx, oropharynx, and vertical facial pattern (numerical values) were evaluated by the Concordance Correlation Coefficient test. 

### Statistical analysis

Data was processed in the statistical program Stata v. 12 (Stata Corp. Texas, USA). Means, standard deviations, minimum and maximum values ​​were calculated. Before making any group comparisons, compliance with the assumptions of normality and homogeneity of variances with Shapiro-Wilk and Levene’s test were evaluated. Some groups were not normally distributed; therefore, Kruskal-Wallis test and U-Mann-Whitney test for pairwise comparisons were applied in them. To compare the means in the groups that met the assumptions, an ANOVA test was used. Comparisons between men and women were performed using Student’s *t* test for independent groups. An analysis of multiple linear regression, in which age and sex were included as factors, was used. Statistical significance was set at 5% in all tests.

## RESULTS

Reliability was considered adequate. High concordance was found, with values greater than 0.93 (Table 1).

**Table 1:** Intra and inter-examiners reliability of the cephalometric tracings for nasopharynx, oropharynx, and facial pattern (quantitative values) [n=24].

Variable	Calibration	CCC	SE	95% CI	Precision	Accuracy	p value
Nasopharynx	Intra-examiner	0.997	0.001	[0.994 - 0.999]	0.997	1.000	<0.001
	Inter-examiners	0.995	0.002	[0.991 - 0.999]	0.996	0.999	<0.001
Oropharynx	Intra-examiner	0.998	0.001	[0.996 - 1.000]	0.998	1.000	<0.001
	Inter-examiners	0.996	0.002	[0.993 - 0.999]	0.997	0.999	<0.001
Facial pattern (quantitative values)	Intra-examiner	0.989	0.001	[0.979 - 0.998]	0.989	0.999	<0.001
	Inter-examiners	0.970	0.002	[0.946 - 0.994]	0.974	0.996	<0.001

CCC, Concordance correlation coefficient; SE, Standard error; CI, Confidence interval.

From lateral cephalograms of 99 Class I patients (considering the ANB angle), according to the facial pattern: 31 were mesofacial (16 to 21 years, 17.67 ± 1.78), 33 dolichofacial (16 to 22 years, 18.68 ± 1.87) and 35 brachyfacial (16 to 21 years, 18.26 ± 1.54).

When comparing upper airway widths in subjects with different facial patterns, statistically significant differences in the nasopharynx where found. Subjects with brachyfacial pattern presented larger airway widths than subjects with mesofacial pattern (*p*= 0.030) and dolichofacial (*p*= 0.034). In the oropharynx no statistically significant differences were found (Table 2).

**Table 2:** Comparison of airway dimensions in subjects with different vertical facial patterns.

Airway	Vertical facial pattern	n	Mean (mm)	SD	Minimum	Maximum	p value*
Nasopharynx	Brachyfacial	35	9.84^ab^	2.71	5.60	18.30	0.043
	Mesofacial	31	8.41^a^	2.14	4.30	12.70	
	Dolichofacial	33	8.42^b^	2.58	3.90	15.00	
	Brachyfacial	35	12.40	3.04	6.70	20.60	
Oropharynx	Mesofacial	31	12.22	2.24	8.80	16.40	0.971
	Dolichofacial	33	12.50	3.30	5.90	18.60	

*Kruskal-Wallis test. a,b: same letters indicate differences, U-Mann-Whitney (a: p=0.034; b: p=0.030); SD: standard deviation.

Table 3 shows upper airway widths according to the different facial patterns compared by sex. There were no statistically significant differences in the nasopharyngeal widths (*p*> 0.05), but statistically significant differences in the oropharynx of brachyfacial and dolichofacial patterns were found (*p*< 0.05). In both, brachyfacial and dolichofacial patterns, oropharynx average width for females was minor than for males (*p*< 0.05).

**Table 3:** Comparison of airway dimensions between females and males with different vertical facial patterns.

Vertical facial pattern*	Airway	n	Female			Male		t	p value
			Mean	SD	n	Mean	SD		
Brachyfacial	Nasopharynx	19	9.53	2.23	16	10.20	3.24	-0.72	0.238
	Oropharynx	19	11.44	2.78	16	13.54	3.01	-2.14	0.020
Mesofacial	Nasopharynx	19	8.45	2.22	12	8.33	2.11	0.15	0.441
	Oropharynx	19	11.91	2.09	12	12.71	2.47	-0.97	0.171
Dolichofacial	Nasopharynx	14	7.86	2.86	19	8.84	2.35	-1.08	0.144
	Oropharynx	14	10.97	2.54	19	13.63	3.40	-2.46	0.010
Total	Nasopharynx	52	8.69	2.46	47	9.17	2.69	-0.93	0.178
	Oropharynx	52	11.48	2.46	47	13.36	3.02	-3.41	0.001

*ANOVA: for nasopharynx; Female, F=2.07, p=0.14; Male, F=1.98, p=0.15; for Oropharynx; Female, F=0.58, p=0.57; Male, F=0.37, p=0.69.

There was no difference between the nasopharyngeal widths of males and females; therefore, the data was combined to compare the widths of the nasopharynx by facial pattern. Data were not combined in the case of the oropharyngeal widths, as sex differences were noted. Table 4 shows the results from the multiple linear regression analysis, separated for the nasopharynx and oropharynx (both considered as continuous variables in the analysis), in which age and sex were included as independent variables. It was observed that facial pattern was associated with nasopharyngeal widths (R^2^= 26.2%, *p*< 0.001). The larger the nasopharyngeal widths, the higher the correlation with the facial pattern. The associated coefficient of variance was relatively low as it explained only around 25% of the total variance. 

**Table 4:** Values of multiple linear regression applied to the airways with the vertical facial pattern (as a continuous variable), age and sex.

Variables		Coefficient	p value	Beta	R^2^
Dependent	Independent				
Nasopharynx	Oropharynx	0.43	<0.001	0.48	0.262*
	Facial pattern	0.48	0.020	0.22	
	Age	-0.11	0.427	-0.07	
	Sex	-0.24	0.618	-0.05	
	Constant	5.62	0.034		
Oropharynx	Nasopharynx	0.51	<0.001	0.45	0.302*
	Facial pattern	-0.24	0.302	-0.09	
	Age	0.03	0.847	0.02	
	Sex	1.60	0.002	0.28	
	Constant	6.58	0.023		

*p < 0.001; Beta, beta function probability distribution; R^2^, coefficient of determination.

## DISCUSSION

An increased interest in upper airway dimensions has been noted lately due to its important role during breathing. Upper airway has been associated to craniofacial complex growth.^1^ ^,^ ^2^ Changes in the normal function of the upper airway during the active period of facial growth could potentially influence craniofacial development.^15^ ^,^ ^16^ However, it is not clear if an altered craniofacial growth pattern might in itself affect upper airway size and therefore facilitate an altered breathing function.

Cephalometric analysis is of great importance to evaluate craniofacial growth pattern both for diagnosis and planning of orthodontic treatment, it is also crucial for communication among professionals; but cephalometric studies often present different interpretations on the description of vertical facial types, which may lead to distinct therapeutic approaches and thus different results.^29^ The Vert index, cephalometrically, distinguishes balanced facial growth (mesofacial), predominance of horizontal facial growth (brachyfacial) and predominance of vertical facial growth (dolichofacial) by using measures related to the growth direction of the mandible. This method has showed to be reliable when compared to the photometric method,^30^ helping to avoid differences between facial bone and soft tissues characteristics, since it was found that hard tissues influence the positioning of soft tissues.^31^

In the present study it was found that brachyfacial patterns had larger anteroposterior linear nasopharyngeal widths in comparison to other vertical facial patterns. These results agree with those reported by Freitas et al,^12^ who found that with a larger vertical pattern, an increased narrowing of the upper airway is expected. Similarly, Ucar et al^15^ found statistically significant differences between low angle and normal angle facial growth for nasopharyngeal airway space, palatal tongue space, upper posterior airway space, but no significant differences in the oropharyngeal airway widths, similar to the present study. These results may suggest that upper airway linear widths could be influenced by the craniofacial growth pattern, especially in brachyfacial individuals. This has been reported before.^32^ The current sample is the largest analyzed so far.

In addition, Ceylan and Oktay^33^ reported that changes in the ANB angle affected nasopharyngeal airway size, and that the oropharyngeal space was reduced in subjects with an enlarged ANB angle. Also a retrusive chin, steep mandibular plane, vertical direction of growth and a tendency toward Class II malocclusion could affect the airway dimensions.^34^ In the present study, only skeletal Class I individuals were included.

Increased nasopharyngeal linear widths in brachyfacial pattern, in comparison to other vertical facial patterns, might be the result of a deficient anteroposterior development of the craniomaxillary complex in brachyfacial pattern.^35^ Facial growth changes may also be related to differences in the direction of condylar growth, and may result from differences in development of anterior facial height and posterior facial height.^36^ These differences in vertical development may lead to rotational growth or positional changes of the mandible, which could affect the airway dimensions. The problem with this hypothesis is that the mandibular positional changes are more likely to affect the oropharynx than the nasopharynx.

Although the findings of the present study do not suggest that patients with brachyfacial pattern have narrower nasopharyngeal widths, this should not be directly linked to a lower frequency of nasal obstruction, even though mouth breathing has been previously related to nasopharyngeal width.^37^ Hypothetically, the narrower the nasopharynx, the less adenoid enlargement would be needed to partially obstruct the nasopharyngeal airway. To the best of our knowledge there has not been any previous study that has associated adenoidal hypertrophy airway obstruction with specific craniofacial patterns. 

In the present study the width of the nasopharynx was measured linearly from a point of the posterior wall of the palate to the posterior pharyngeal wall, where there was an apparent reduction of the airway. This measurement was below the anatomical location of adenoidal tissues. Adenoid hypertrophy is the most common cause of nasopharyngeal obstruction in children and the most common cause of pediatric sleep disordered breathing, which mounting emerging evidence continues to suggest the need for its multidisciplinary management.^38^

It is possible to mention two study limitations: its retrospective characteristics and because bidimensional images were considered. Because of the retrospective nature of the study, a direct assessment of the breathing pattern for each patient was not possible. Only information from the clinical charts was used to rule out the presence or not of pharyngeal obstructive pathology. The selected patients had no history of pharyngeal pathology, chronicity of nasal and airway patency, smoking status, obesity and use of medications which have been suggested to have influence on airway dimensions; consequently, they were assumed to have a healthy pharyngeal function, but this proxy strategy is questionable as it may not likely detect mild to moderate pharyngeal obstructions,^39^ however the same criteria of head and mandibular position were applied to all groups, so any misclassification problem should have been distributed evenly in all the analyzed groups. The current sample is twice the minimal required size as a means to reduce the potential impact of this limitation. Therefore, because only relatively healthy pharyngeal patients with malocclusions were selected, we expected that the pharyngeal widths should only reflect natural anatomical variations when no major pharyngeal pathology was present.

To eliminate the potential influence of growth and aging, only post-pubertal subjects were selected for the current study. Lymphoid tissues are known to vary significantly during craniofacial growth. After puberty their size should be approximately normal. 

This study was performed with two-dimensional cephalometric films that evaluate only pharyngeal airway linear width. A more comprehensive three-dimensional evaluation would have required an ENT assessment and more complex otorhinolaryngology equipment.^40^

Lateral cephalometry offers only a 2-dimensional illustration of a 3-dimensional structure, and there are studies that have claimed that inaccurate determination of the airway size may lead to unreliable results^16^ and sagittal linear measurements used are weakly correlated with cross sectional area measurements in CBCT, which would be more important to describe airway patency;^41^ ^,^ ^42^ but a recently publish systematic review concluded that no ideal diagnostic tool exists currently for dentists to reliably screen, particularly, adenoid hypertrophy.^43^ More research to identify a low-risk, easily acceptable, highly valid diagnostic screening tool is suggested. Nevertheless, despite the use of cephalometric films, with its known limitations, the findings of the present study suggest that not only dolichofacial, but also mesofacial individuals may need to be carefully screened for potential limited pharyngeal dimensions. 

Finally, the association found between pharyngeal width and facial pattern suggests that in clinical practice orthodontic, orthopedic, and orthognathic treatments should be oriented to prevent the reduction of nasopharyngeal anteroposterior widths, or even help to increase them, mainly in dolichofacial and mesofacial individuals.

## CONCLUSIONS

Nasopharyngeal anteroposterior linear widths in skeletal Class I malocclusion brachyfacial individuals are larger than in mesofacial and dolichofacial individuals. No differences were noted for the oropharyngeal widths.

Although a positive correlation between nasopharyngeal widths and vertical facial pattern was found, the Vert index only explained 25% of the total variability. No significant correlation was found for the oropharyngeal widths.

Based on the results, treatments should be oriented to prevent the reduction of nasopharyngeal anteroposterior width, or even help to increase them, mainly in dolichofacial and mesofacial individuals.
